# Formetric 4D Rasterstereography

**DOI:** 10.1155/2014/315041

**Published:** 2014-03-31

**Authors:** Johnny Padulo, Luca Paolo Ardigò

**Affiliations:** ^1^Tunisian Research Laboratory “Sports Performance Optimization”, National Center of Medicine and Science in Sport, 263 Tunis, Tunisia; ^2^School of Exercise and Sport Science, Department of Neurological and Movement Sciences, University of Verona, Via Felice Casorati, No. 43, 37131 Verona, Italy

This paper is a comment on “Intra- and interday reliability of spine rasterstereography” [1]. We think that supporting the feasibility of using the rasterstereographic tool Formetric 4D, both as a diagnostic tool and in scientific research, requires much more scientific exactness. The purpose of this paper is to underline what we consider to be substantial omission or error.

In research, validity is the extent to which a conclusion corresponds accurately to the reality. Differently, reliability is the overall consistency of a measure [2]. Validity means a high level of accuracy, while reliability means a high level of precision [3]. The conclusions of the paper are supported by the results in terms of reliability (“In conclusion, the present study reveals a good to great reliability of the DIERS Formetric 4D system depending on the typology of the measured parameter.”) but not in terms of validity [4–6] (“Therefore, this study validated aspects of the rasterstereographic measuring system that potentially could replace X-rays in follow-up of spinal deformities helping to reduce X-rays irradiation.”) [1]. A clarification should be done in this paper to better explain the state of the art about the topic described in this paper.

In conclusion, “Therefore, this study validated aspects of the rasterstereographic measuring system that potentially could replace X-rays in follow-up of spinal deformities helping to reduce X-rays irradiation” [1]. Discussion should provide only those conclusions that are supported by the study [7]. At present, Formetric 4D cannot be considered validated but only reliable. Further evidence-based research is necessary to support Formetric 4D effectiveness.

We should verify the data from this study with caution before suggesting such a clear indication [8, 9]. Attention of clinical researchers should focus on patients' needs. Patients deserve both examination validity and reliability, and reduced exposure to ionizing radiation. Firstly, a minimum acceptable examination validity and reliability level has to be established. Then, researchers should investigate alternative methodologies capable of achieving at least that specific level. Sound research on this topic is continuously needed.
